# PPCM: Combing Multiple Classifiers to Improve Protein-Protein Interaction Prediction

**DOI:** 10.1155/2015/608042

**Published:** 2015-10-11

**Authors:** Jianzhuang Yao, Hong Guo, Xiaohan Yang

**Affiliations:** ^1^Department of Biochemistry and Cellular and Molecular Biology, University of Tennessee, Knoxville, TN 37996, USA; ^2^Biosciences Division, Oak Ridge National Laboratory, Oak Ridge, TN 37831, USA

## Abstract

Determining protein-protein interaction (PPI) in biological systems is of considerable importance, and prediction of PPI has become a popular research area. Although different classifiers have been developed for PPI prediction, no single classifier seems to be able to predict PPI with high confidence. We postulated that by combining individual classifiers the accuracy of PPI prediction could be improved. We developed a method called protein-protein interaction prediction classifiers merger (PPCM), and this method combines output from two PPI prediction tools, GO2PPI and Phyloprof, using Random Forests algorithm. The performance of PPCM was tested by area under the curve (AUC) using an assembled Gold Standard database that contains both positive and negative PPI pairs. Our AUC test showed that PPCM significantly improved the PPI prediction accuracy over the corresponding individual classifiers. We found that additional classifiers incorporated into PPCM could lead to further improvement in the PPI prediction accuracy. Furthermore, cross species PPCM could achieve competitive and even better prediction accuracy compared to the single species PPCM. This study established a robust pipeline for PPI prediction by integrating multiple classifiers using Random Forests algorithm. This pipeline will be useful for predicting PPI in nonmodel species.

## 1. Introduction

Protein-protein interaction (PPI) networks play important roles in many cellular activities, including complex formation and metabolic pathways [1], and identification of PPI pairs may provide important insights into the molecular basis of cellular processes [2]. Several high-throughput experimental approaches have been developed for PPI identification, including two-hybrid assays [3], tandem affinity purification followed by Mass Spectrometry [4], and protein microarrays [5]. These high-throughput methods have produced a large amount of PPI data, which have been accumulated in the public PPI databases, such as DIP [6] and STRING [7]. However, the results generated by these high-throughput methods may lack reliability [8] and have limited coverage of PPIs in any given organism [9]. Additional experimental information for PPI is also available, including the X-ray structures of protein complexes in the PDB databank [10]. Nevertheless, the information from protein structure complexes may be limited compared to the large volume of protein sequences available in the public databases [11].

To overcome the limitations in PPI identification using experimental methods, computational approaches have been developed to achieve large-scale PPI prediction in various organisms [12–17]. Traditional input features for PPI prediction are mainly from biological data sources, which may be divided into four categories: Gene Ontology- (GO-) based, structure-based, network topology-based, and sequence-based features [18]. Each individual computational PPI prediction method utilizes only one or few input sources for PPI prediction. For example, BIPS only takes protein sequences as input for Interolog searching [19]. Bio::Homology::InterologWalk takes protein sequences and well-known PPI networks as input [12]. Although these methods using single or several features as input can generate fairly accurate results, they are unable to take advantage of other input features that could be helpful for PPI prediction. Thus, machine learning methods (e.g., Bayesian classifiers [20], Artificial Neural Networks (ANN) [21], Support Vector Machines (SVM) [22], and Random Forests [23]) have been developed to integrate multiple features as inputs. Machine learning approaches have shown better performances compared to some other methods; among them, Random Forests method seems to show the best performance [24]. In addition, PPI prediction is associated with imbalanced data problem. Zhang et al. [25] proved that the imbalanced data problem could be solved by ensemble methods. Augusty and Izudheen [26] further showed that Random Forests method could improve Zhang's methods in dealing with the imbalanced data problem.

In addition to the progress in identification of informative features for PPI prediction, a variety of algorithms have been developed to improve the PPI prediction accuracy [18]. For instance, Phylogenetic Profiling (PP) uses genome-scale and network-based features as inputs for PPI prediction founded on the assumption that the cooccurrence of two proteins across taxa indicates a good chance for them to function together [27, 28]. Although PPI prediction by PP has shown good performance in prokaryotes, it has poor performance in PPI prediction in eukaryotes, probably due to modularity of eukaryotic proteins, biased diversity of available genomes, and large evolutionary distances [29, 30]. Several studies indicate that the accuracy of PPI prediction by PP can be improved by selecting the appropriate reference taxa and matching the reference taxa to the known PPI network [30–32]. Recently, Simonsen et al. developed a PPI prediction software Phyloprof [33] that integrates four PPI prediction methods including the original PP method [27], mutual information (MI) method [34], hypergeometric distribution based method [35], and the extension of the hypergeometric distribution (RUN) method [36]. Also, Phyloprof provides six reference taxa optimization methods including Tree Level Filtering, Iterative Taxon Selection, Genetic Algorithm, and Tree based search [33, 37]. Furthermore, there are four PPI networks available in Phyloprof, including the networks from* Escherichia coli* (EC),* Saccharomyces cerevisiae* (hereafter referred to as SC),* Drosophila melanogaster* (DM), and* Arabidopsis thaliana* (AT). In short, Phyloprof provides a series of PPI prediction classifiers as a result of various combinations of PPI prediction methods, reference taxa optimization methods, and networks from different species.

Another sophisticated PPI prediction software called GO2PPI has been developed to use Gene Ontology and PPI networks as input [38]. By introducing a concept called inducer to combine machine learning and semantic similarity techniques, GO2PPI can provide a series of PPI prediction classifiers that are combinations of machine learning methods (i.e., Naïve Bayes (NB) and Random Forests), GO categories (i.e., biological process (BP), cellular component (CC), and molecular function (MF)), and networks from seven species (*Homo sapiens* (HS),* Mus musculus* (MM),* S. pombe* (SP), SC, AT, EC, and DM).

A variety of ensemble classifiers have been proposed in different bioinformatics studies and showed generally better performance than individual classifiers [39–41]. To build on this research, we developed a pipeline PPCM (i.e., PPI prediction classifiers merger) to enhance the PPI prediction accuracy by merging multiple PPI prediction classifiers using Random Forests algorithm. To the best of our knowledge, this study is the first effort to merge multiple classifiers (Phyloprof and GO2PPI) by machine learning for PPI prediction.

## 2. Methods

### 2.1. Construction of a Gold Standard Dataset

We created training and test dataset containing direct interacted protein pairs of yeast for protein-protein interaction (PPI) prediction using a method described by Qi et al. [24]. Briefly, 2865 positive PPI pairs were obtained from the DIP database [6]. These direct interaction protein pairs were tested to be highly confident PPI pairs by small-scale experiments. Since there was insufficient high-confidence negative data [42], negative PPI pairs were generated by randomly pairing proteins followed by removing the positive PPI pairs [43]. Finally, the positive PPI pairs and the negative PPI pairs were combined by a ratio of 1 to 100 into a “Gold Standard” dataset. It has been proved that the AUC value is not sensitive to the different positive-to-negative ratios (e.g., from 1 : 2 to 1 : 100) by both GO2PPI and Phyloprof.

### 2.2. Selection of Features for PPI Prediction

The results of PPI prediction classifiers were used as features of PPCM. Specifically, Phyloprof has three kinds of input parameters, including four PPI prediction methods, eight Reference Taxa Optimization methods, and four PPI networks. Without the time-consuming PPI prediction method “RUN,” there were 96 different classifiers based on different combinations of parameters provided by Phyloprof (Table  S2 in Supplementary Material available online at http://dx.doi.org/10.1155/2015/608042). As mentioned above, GO2PPI has three kinds of input parameters as well, including two machine learning methods, seven GO terms or terms combinations (BP, CC, MF, BPCC, BPMF, CCMF, and BPCCMF), and seven PPI networks. In the same way, there were 98 different combinations of classifiers provided by GO2PPI (Table  S1). We used combined GO terms in this study, because the best accuracy was achieved by the integration of three GO terms in the GO2PPI paper [38].

### 2.3. PPI Prediction Using PPCM Pipeline

**Figure 1:**
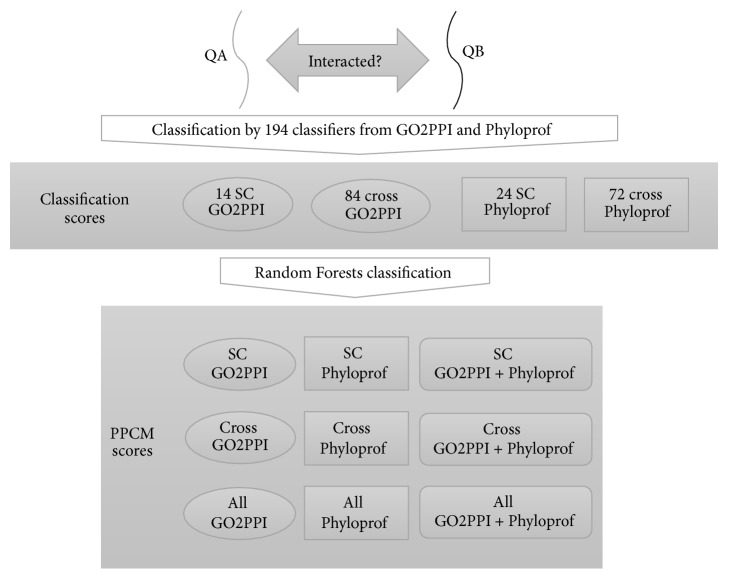
The PPCM pipeline for protein-protein interaction prediction. Given a pair of query proteins QA and QB, their interaction possibility was first predicted by each of the 194 classifiers from GO2PPI and Phyloprof. Then, the classification scores were merged using Random Forests algorithm to generate the final PPI prediction score. Nine PPI classification scores were provided by PPCM. “SC” represents PPI networks in* Saccharomyces cerevisiae*. “Cross” represents all PPI networks except SC. “All” represents all PPI networks in both SC and cross species.

The PPCM pipeline, as illustrated in Figure 1, was developed to combine multiple classifiers for enhancing PPI prediction accuracy. Specifically, a protein pair is first evaluated by classifiers provided by PPI prediction software, such as GO2PPI [38] and Phyloprof [33]. Then, the classification scores from individual classifiers are used as input features to generate the final PPI prediction score using Random Forests algorithm, implemented in the Berkeley Random Forests package [44]. GO2PPI has 98 PPI prediction classifiers, among which 14 are SC-related and 84 are not SC-related (cross species) classifiers (Table  S1). Phyloprof has 96 PPI prediction classifiers, among which 24 are SC-related and 72 are not SC-related (cross species) classifiers (Table  S2).

### 2.4. Evaluation of PPI Prediction Accuracy

The aforementioned Gold Standard database that contains about 30,000 PPI pairs with a positive-to-negative PPI ratio of 1 : 100 was used to evaluate the PPI prediction accuracy. The following measures were used to evaluate PPI prediction results: the true positive rate (TPR, also called sensitivity), defined as the ratio of correctly predicted positive PPI pairs among all positive PPI pairs, the true negative rate (TNR, also called specificity), defined as the ratio of correctly predicted negative PPI pairs among all negative PPI pairs, and the false positive rate (FPR, also called Type I error), defined as the ratio of incorrectly predicted PPI pairs among all negative PPI pairs. FPR is one minus TNR. The receiver operating characteristic (ROC) curves were created by plotting TPR versus FPR. The area under the curve (AUC) was used as a measure of the prediction accuracy. The AUC value was calculated using the following equation:

(1) $$\begin{array}{c} \text{AUC}=\frac{1}{2}\overset{n}{\underset{k=1}{\sum }}\left(\left(X_{k}-X_{k-1}\right)\left(Y_{k}+Y_{k-1}\right)\right), \end{array}$$

where *X*_*k*_ is the FPR at *k* pair and *Y*_*k*_ is the TPR at *k* pair in the ranked PPI pair list.  The prediction process was repeated 25 times, and the average AUC value was reported.

We evaluated the PPI prediction accuracy of PPCMs and the classifiers in GO2PPI and Phyloprof using AUC. We introduced three categories of PPCM, including GO2PPI, Phyloprof, and GO2PPI + Phyloprof, with each further divided to three subcategories: SC, cross species, and all species (i.e., SC plus cross species) (Figure 1).

## 3. Results and Discussion

### 3.1. Performance of PPCM in GO2PPI Category

**Figure 2:**
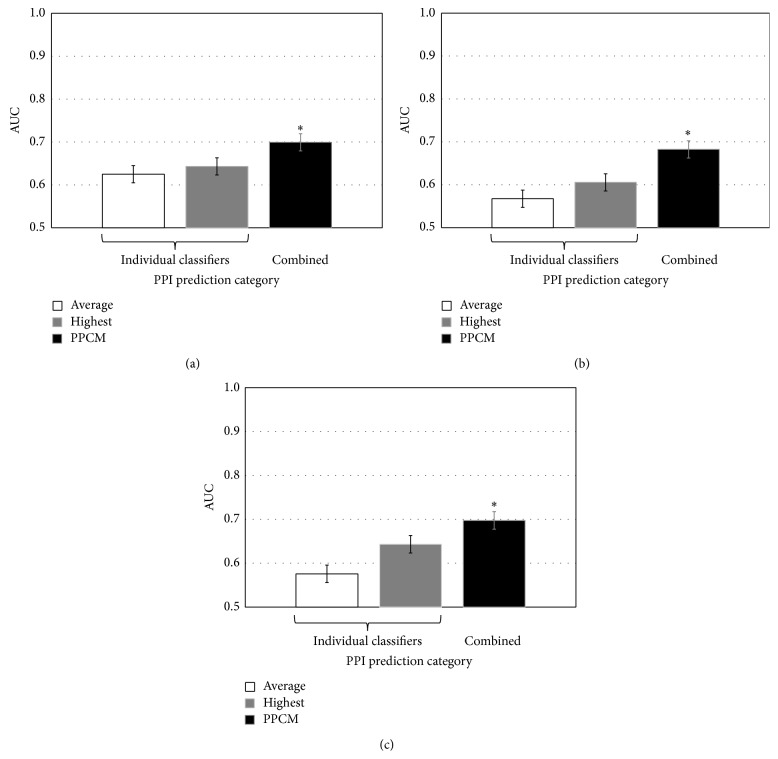
Comparison of PPI prediction accuracy in the GO2PPI category. (a) PPI prediction based on classifiers related to SC. (b) PPI prediction based on classifiers related to cross species. (c) PPI prediction based on classifiers related to all species. “Average” represents the mean AUC of all the classifiers in each category. “Highest” represents the classifier with highest AUC among all the classifiers in each category. Error bars show standard deviation. “*∗*” indicates that AUC of PPCM was significantly (*P* value < 0.05; *t*-test) higher than that of the most accurate classifier in each category.

Using our Gold Standard dataset, the average AUC of the 14 SC-related classifiers in GO2PPI (Table  S1) was 0.63 and rf|bpcc|SC was the most accurate classifier, with an AUC of 0.64, among these 14 classifiers (Figure 2(a)). The average AUC of the 84 cross species related classifiers in GO2PPI (Table  S1) was 0.57 and rf|bpcc|HS was the most accurate classifier, with an AUC of 0.61, among these 84 classifiers (Figure 2(b)). The average AUC of all the 96 (all species) classifiers in GO2PPI (Table  S1) was 0.58 and rf|bpcc|SC was the most accurate classifier, with an AUC of 0.64, among these 98 classifiers (Figure 2(c)). The AUCs of PPCMs are 0.70, 0.68, and 0.70 for SC, cross species, and all species PPCM, respectively (Figure 2). These results indicate that PPCMs significantly improved PPI prediction accuracy compared with their corresponding classifiers in GO2PPI category.

Compared with the most accurate classifier in GO2PPI category, the cross species PPCM improves AUC by 11%. The improvement of PPCM in SC PPCM was only 9% (Figure 2), indicating that the cross species PPCM had better performance than the SC classifier. The better performance of cross species PPCM (containing 84 features) than SC PPCM (containing 14 features) suggests that the larger number of features incorporated into PPCM enhanced PPI prediction accuracy in GO2PPI category.

### 3.2. Performance of PPCM in the Phyloprof Category

**Figure 3:**
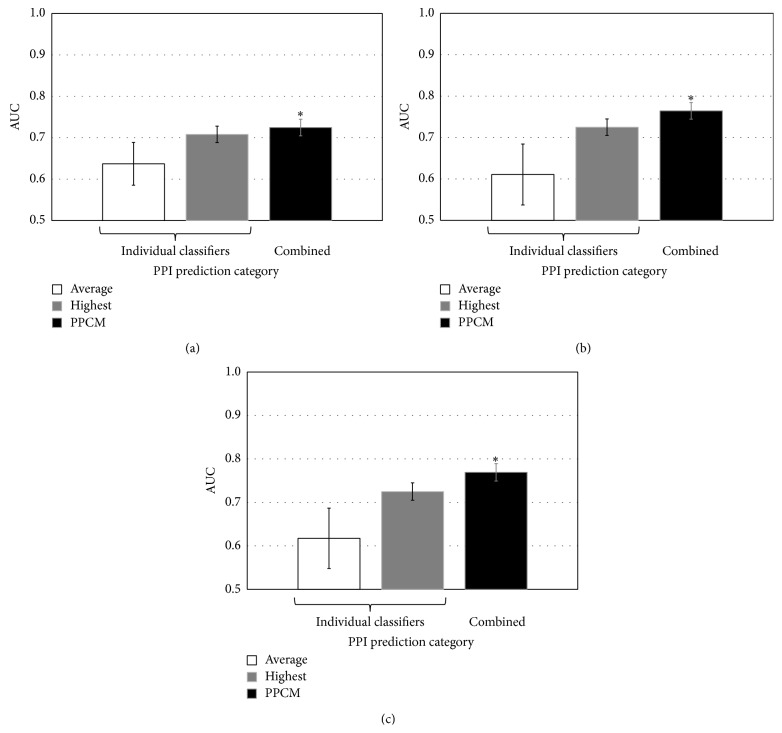
Comparison of PPI prediction accuracy in the Phyloprof category. (a) PPI prediction based on classifiers related to SC. (b) PPI prediction based on classifiers related to cross species. (c) PPI prediction based on classifiers related to all species. “Average” represents the mean AUC of all the classifiers in each category. “Highest” represents the classifier with highest AUC among all the classifiers in each category. Error bars show standard deviation. “*∗*” indicates that AUC of PPCM was significantly (*P* value < 0.05; *t*-test) higher than that of the most accurate classifier in each category.

Again, using our Gold Standard dataset, the average AUC of the 24 SC-related classifiers in Phyloprof (Table  S2) was 0.64 and SC|mi|et was the most accurate classifier, with an AUC of 0.71, among these 24 classifiers (Figure 3(a)). The average AUC of the 72 cross species related classifiers in Phyloprof (Table  S2) was 0.61 and EC|mi|et was the most accurate classifier, with an AUC of 0.72, among these 84 classifiers (Figure 3(b)). The average AUC of all the 96 (all species) classifiers in Phyloprof (Table  S2) was 0.62 and mi|et|EC was the most accurate classifier, with an AUC of 0.72, among these 96 classifiers (Figure 3(c)). The AUCs of PPCMs are 0.72, 0.76, and 0.77 for SC, cross species, and all species PPCM, respectively (Figure 3). These results indicate that PPCMs significantly improved PPI prediction accuracy compared with their corresponding classifiers in the Phyloprof category. Compared with the most accurate classifier in the Phyloprof category, the cross species PPCM improves AUC by 6%, while the improvement by SC PPCM is only 1% (Figure 3), indicating that the cross species PPCM had better performance in AUC improvement. The better performance of cross species PPCM (containing 72 features) than SC PPCM (containing 24 features) suggests that more features incorporated into PPCM could enhance PPI prediction accuracy in the Phyloprof category.

### 3.3. Performance of PPCM in GO2PPI + Phyloprof Category

**Figure 4:**
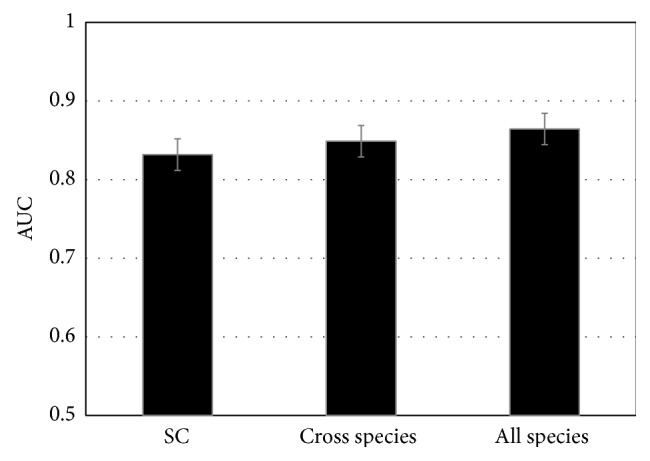
Comparison of PPI prediction accuracy in the GO2PPI + Phyloprof category. Error bars show standard deviation.

After separate evaluation of PPCM in the GO2PPI and Phyloprof categories, we assessed the performance of PPCM in the GO2PPI + Phyloprof category which combined all the classifiers in both GO2PPI and Phyloprof. The AUCs of PPCMs in the GO2PPI + Phyloprof category were 0.83, 0.85, and 0.86 for SC, cross species, and all species PPCM, respectively (Figure 4), which are significantly higher than those of PPCMs in either GO2PPI or Phyloprof category separately (Figures 2 and 3). Compared with the highest AUCs of individual classifiers in GO2PPI and Phyloprof category, the cross species PPCM improves AUC by 18% and the improvement by SC PPCM was 17% (Figures 2, 3, and 4). These results indicate that PPCM based on all the 194 classifiers from both GO2PPI and Phyloprof could generate more accurate PPI prediction than PPCM based on a fewer number of classifiers in GO2PPI or Phyloprof individually, further supporting the aforementioned premise that more features incorporated into PPCM would enhance PPI prediction accuracy. In summation, based on our combinatorial approach, our cross species PPCM results yield informative predictions that will help build high-quality PPI networks for nonmodel organisms. Such prediction will be valuable for nonmodel organisms that lack biological data and PPI prediction software for nonmodel organisms [18].

Recently, ensemble classifiers, for example, LibD3C, were developed based on a clustering and dynamic selection strategy [39]. In order to compare the performance of Random Forests method applied by our PPCM with the latest ensemble classifiers, we performed ensemble classifiers calculation on our all species training and testing datasets of the GO2PPI  +  Phyloprof category (see Figure 4) by LibD3C in Weka-3.7.12 with default setting. The average AUC by LibD3C was 0.86 ± 0.03 which is in an excellent agreement with our Random Forests result (0.86 ± 0.02). Therefore, Random Forests method applied by our PPCM shows very similar performance with the latest ensemble classifiers (LibD3C).

## Supplementary Material

Supplementary Table S1. lists the classifiers of GO2PPI.
Supplementary Table S2. lists the classifiers of Phyloprof.
