# Supplementary material for: PPCM: Combing Multiple Classifiers to Improve Protein-Protein Interaction Prediction
Source: Int J Genomics. 2015 Oct 11;2015:608042. doi: 10.1155/2015/608042 (PMC4619929; doi:10.1155/2015/608042)
Supplement: Supplementary file 1 — Supplementary Table S1. lists the classifiers of GO2PPI. Supplementary Table S2. lists the classifiers of Phyloprof. [file 608042.f1.pdf]

Table S1. The individual classifiers of GO2PPI merged by PPCM

NO.	Machine learning algorithm|GO|NetWork
1	nb|bp|AT
2	nb|bp|DM
3	nb|bp|EC
4	nb|bp|HS
5	nb|bp|MM
6	nb|bp|SC
7	nb|bp|SP
8	nb|bpcc|AT
9	nb|bpcc|DM
10	nb|bpcc|EC
11	nb|bpcc|HS
12	nb|bpcc|MM
13	nb|bpcc|SC
14	nb|bpcc|SP
15	nb|bpccmf|AT
16	nb|bpccmf|DM
17	nb|bpccmf|EC
18	nb|bpccmf|HS
19	nb|bpccmf|MM
20	nb|bpccmf|SC
21	nb|bpccmf|SP
22	nb|bpmf|AT
23	nb|bpmf|DM
24	nb|bpmf|EC
25	nb|bpmf|HS
26	nb|bpmf|MM
27	nb|bpmf|SC
28	nb|bpmf|SP
29	nb|cc|AT
30	nb|cc|DM
31	nb|cc|EC
32	nb|cc|HS
33	nb|cc|MM
34	nb|cc|SC
35	nb|cc|SP
36	nb|ccmf|AT
37	nb|ccmf|DM

38	nb|ccmf|EC
39	nb|ccmf|HS
40	nb|ccmf|MM
41	nb|ccmf|SC
42	nb|ccmf|SP
43	nb|mf|AT
44	nb|mf|DM
45	nb|mf|EC
46	nb|mf|HS
47	nb|mf|MM
48	nb|mf|SC
49	nb|mf|SP
50	rf|bp|AT
51	rf|bp|DM
52	rf|bp|EC
53	rf|bp|HS
54	rf|bp|MM
55	rf|bp|SC
56	rf|bp|SP
57	rf|bpcc|AT
58	rf|bpcc|DM
59	rf|bpcc|EC
60	rf|bpcc|HS
61	rf|bpcc|MM
62	rf|bpcc|SC
63	rf|bpcc|SP
64	rf|bpccmf|AT
65	rf|bpccmf|DM
66	rf|bpccmf|EC
67	rf|bpccmf|HS
68	rf|bpccmf|MM
69	rf|bpccmf|SC
70	rf|bpccmf|SP
71	rf|bpmf|AT
72	rf|bpmf|DM
73	rf|bpmf|EC
74	rf|bpmf|HS
75	rf|bpmf|MM
76	rf|bpmf|SC
77	rf|bpmf|SP
78	rf|cc|AT

79	rf|cc|DM
80	rf|cc|EC
81	rf|cc|HS
82	rf|cc|MM
83	rf|cc|SC
84	rf|cc|SP
85	rf|ccmf|AT
86	rf|ccmf|DM
87	rf|ccmf|EC
88	rf|ccmf|HS
89	rf|ccmf|MM
90	rf|ccmf|SC
91	rf|ccmf|SP
92	rf|mf|AT
93	rf|mf|DM
94	rf|mf|EC
95	rf|mf|HS
96	rf|mf|MM
97	rf|mf|SC
98	rf|mf|SP

Table S2. The individual classifiers of Phyloprof merged by PPCM.

NO.	Network|taxa optimization|PPI prediction
1	AT|hg|et
2	AT|hg|ga
3	AT|hg|gapd
4	AT|hg|gatc
5	AT|hg|ite
6	AT|hg|ot
7	AT|hg|rt
8	AT|hg|to
9	AT|mi|et
10	AT|mi|ga
11	AT|mi|gapd
12	AT|mi|gatc
13	AT|mi|ite
14	AT|mi|ot
15	AT|mi|rt
16	AT|mi|to
17	AT|sb|et
18	AT|sb|ga
19	AT|sb|gapd
20	AT|sb|gatc
21	AT|sb|ite
22	AT|sb|ot
23	AT|sb|rt
24	AT|sb|to
25	SC|hg|et
26	SC|hg|ga
27	SC|hg|gapd
28	SC|hg|gatc
29	SC|hg|ite
30	SC|hg|ot
31	SC|hg|rt
32	SC|hg|to
33	SC|mi|et
34	SC|mi|ga
35	SC|mi|gapd
36	SC|mi|gatc
37	SC|mi|ite
38	SC|mi|ot
39	SC|mi|rt

40	SC|mi|to
41	SC|sb|et
42	SC|sb|ga
43	SC|sb|gapd
44	SC|sb|gatc
45	SC|sb|ite
46	SC|sb|ot
47	SC|sb|rt
48	SC|sb|to
49	HS|hg|et
50	HS|hg|ga
51	HS|hg|gapd
52	HS|hg|gatc
53	HS|hg|ite
54	HS|hg|ot
55	HS|hg|rt
56	HS|hg|to
57	HS|mi|et
58	HS|mi|ga
59	HS|mi|gapd
60	HS|mi|gatc
61	HS|mi|ite
62	HS|mi|ot
63	HS|mi|rt
64	HS|mi|to
65	HS|sb|et
66	HS|sb|ga
67	HS|sb|gapd
68	HS|sb|gatc
69	HS|sb|ite
70	HS|sb|ot
71	HS|sb|rt
72	HS|sb|to
73	EC|hg|et
74	EC|hg|ga
75	EC|hg|gapd
76	EC|hg|gatc
77	EC|hg|ite
78	EC|hg|ot
79	EC|hg|rt
80	EC|hg|to

81	EC|mi|et
82	EC|mi|ga
83	EC|mi|gapd
84	EC|mi|gatc
85	EC|mi|ite
86	EC|mi|ot
87	EC|mi|rt
88	EC|mi|to
89	EC|sb|et
90	EC|sb|ga
91	EC|sb|gapd
92	EC|sb|gatc
93	EC|sb|ite
94	EC|sb|ot
95	EC|sb|rt
96	EC|sb|to
